# Supplementary figures and images for: Combining serological and contact data to derive target immunity levels for achieving and maintaining measles elimination
Source: BMC Med. 2019 Sep 25;17:180. doi: 10.1186/s12916-019-1413-7 (PMC6760101; doi:10.1186/s12916-019-1413-7)

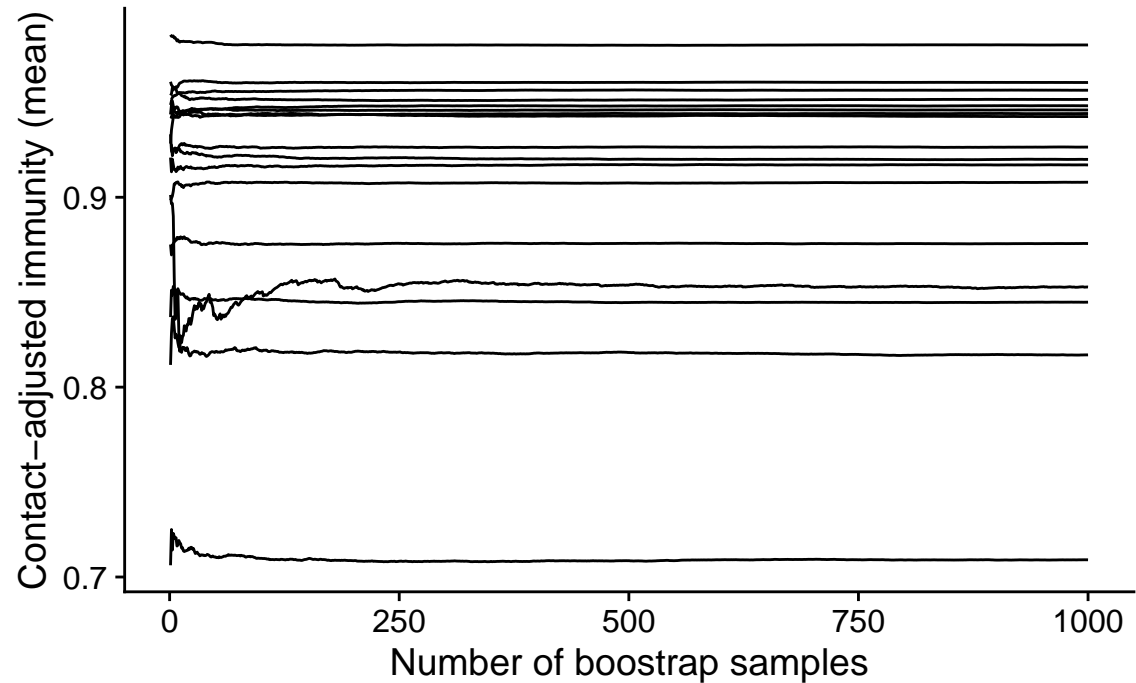

Supplement: Supplementary file 1 — Supplementary Figure 1. Mean estimated of contact-adjusted immunity as a function of the number of bootstrap samples. Each line represents one country. (PDF 69 kb) [file 12916_2019_1413_MOESM1_ESM.pdf]
